# Phenomenology and Outcomes of In‐Patients With Parkinson's Disease During the Coronavirus Disease 2019 Pandemic

**DOI:** 10.1002/mds.28205

**Published:** 2020-07-31

**Authors:** Christopher Kobylecki, Theodora Jones, Cheng Khuang Lim, Carol Miller, Alexander M. Thomson

**Affiliations:** ^1^ Department of Neurology, Manchester Centre for Clinical Neurosciences Salford Royal NHS Foundation Trust Salford United Kingdom; ^2^ Manchester Academic Health Sciences Centre University of Manchester Manchester United Kingdom; ^3^ Department of Ageing and Complex Medicine Salford Royal NHS Foundation Trust Salford United Kingdom

The early months of 2020 saw the emergence of a novel coronavirus infection (severe acute respiratory syndrome coronavirus 2), which rapidly developed into a global pandemic.^1^ Coronavirus disease 2019 (COVID‐19) illness is characterized by viral pneumonitis and in severe cases acute respiratory distress syndrome and death.^2^ Key risk factors for disease severity and mortality include advancing age, male sex, ethnicity, diabetes mellitus, and hypertension.^2^

**TABLE 1:** Demographic features and comorbidities of in‐patients with idiopathic PD dying in pandemic and control cohorts

Characteristic	Control Cohort	Pandemic Cohort	*P* Value, Odds Ratio (95% CI)
Age	79.8 (8.5)	78.3 (9.5)	0.6
Sex	15 males, 12 females	8 males, 5 females	1.0
Deceased	27/414 (6.5%)	13/58 (22%)	**0.0003, 4.1 (2.0–8.6)**
Dementia	7/27 (26%)	7/13 (54%)	0.15, 3.3 (0.8**–**13.4)
Delirium	21/27 (78%)	9/13 (69%)	0.7, 0.6 (0.2**–**2.8)
Diabetes	8/27 (30%)	1/13 (8%)	0.23, 0.2 (0.0**–**1.8)
Hypertension	10/27 (37%)	5/13 (38%)	1.0, 1.1 (0.3**–**4.2)
COVID‐19 infection	n/a	Confirmed 3 Probable 1	
Cause of death	Pneumonia 13 Cardiac 4 COPD 3 Other 7	COVID‐19 Pneumonia 3 Pneumonia 3 PD related 3 Cardiac 1 Other 3	

Data are presented as mean (standard deviation) or number (percentage). Bold text indicates statistically significant (*P* < 0.05).

CI, confidence interval; COVID‐19, coronavirus disease 2019; COPD, chronic obstructive pulmonary disease; PD, Parkinson's disease.

The impact and outcomes of COVID‐19 disease on people admitted to hospital with idiopathic Parkinson's disease (iPD) are not fully understood. We aimed to evaluate mortality rates and risk factors in hospitalized patients with iPD during the COVID‐19 pandemic.

We evaluated all patients with a known diagnosis of iPD who had an unplanned medical admission to a university teaching hospital between January 1, 2020 and May 31, 2020, known as the pandemic cohort. We chose this time frame corresponding to the international recognition of cases and consistent with definitions used in other studies of COVID‐19 in iPD.^3^ Demographic details including age, sex and ethnicity, the presence of delirium, and comorbidities of dementia, diabetes, and hypertension were recorded. Similar data were collated from controls representing 3 year‐long periods of observational data collection. Elective admissions, those with non‐iPD diagnoses, and patients admitted to nonmedical services such as surgery were excluded. Statistical analysis was performed using Prism 5 (GraphPad, La Jolla, CA). Continuous variables were compared using Student's *t* test and categorical variables using Fisher's exact test.

In the pandemic cohort, 58 in‐patient episodes were identified (Table 1). A total of 13 deaths (22.4%) were recorded, significantly increased compared with 27/414 (6.5%) in the control cohort (*P* = 0.0003). COVID‐19 was confirmed in 3 cases and probable in 1. Two other patients tested positive and survived. There were no differences in mean age or sex distribution in those dying in either cohort. All but 1 deceased patient in each cohort was of white British origin.

There was a nonsignificantly higher proportion of patients with dementia in the pandemic cohort, although no differences in delirium or hypertension. Whereas 30% of patients who died in the control cohort had a diagnosis of diabetes, only 1 in the pandemic cohort, who was COVID negative, had diabetes. This is a notable difference to the population data, which suggest patients with diabetes are at a higher risk of dying from COVID‐19.^2^

Compared with a 3‐year period of control data, we show a higher‐than‐expected rate of deaths in hospitalized patients with iPD during the pandemic period. As well as apparent high mortality in those positive for COVID‐19, indirect effects on mortality in other patients are likely. Delay in seeking medical attention in a pandemic situation is likely to result in more advanced presentations of other conditions. Lack of service capacity to appropriately escalate other non‐COVID‐19–related acute illness and decompensation in parkinsonism leading to greater vulnerability are further potential indirect mechanisms. Across the reported time period, there was no other significant difference in our hospital services, which may have influenced the outcomes seen. There was no bias for seasonal variation in mortality compared with the control group.

This observational study adds to the growing epidemiological data on survival and mortality in COVID‐19 illness. Emerging data suggest a high mortality in patients with advanced iPD and COVID‐19^4^ as well as worsening of motor and nonmotor functions.^3^ Mortality in moderately affected community‐dwelling iPD patients may differ.^5^ These results highlight the vulnerability of patients with PD in hospitals and in the community. This work allows us to start to risk stratify this group and will inform advice on steps to reduce transmission and infection as well as wider public health approaches.

## Author Roles

(1) Research Project: A. Conception, B. Organization, C. Execution; (2) Statistical Analysis: A. Design, B. Execution, C. Review and Critique; (3) Manuscript: A. Writing of the First Draft, B. Review and Critique.

C.K.: 1A, 2A, 2B, 3A, 3B

T.J.: 1A, 1C, 3B

C.K.L.: 1A, 1C, 3B

C.M.: 1A, 1C, 3B

A.M.T.: 1A, 1C, 3A, 3B

## Full financial disclosures for the previous 12 months

C.K. has received grants from Parkinson's UK and the Michael J. Fox Foundation, speaker fees from Britannia and Bial Pharma, support to attend international meetings from Abbvie, and advisory boards from Abbvie. T.J., C.K.L., C.M., and A.M.T. have no financial disclosures to report.
